# Notes and News

**Published:** 1897-06-19

**Authors:** 


### NOTES AND NEWS.

(Collected and Communicated.)

Mr. A. D. Fripp, M.S., has been appointed assistant surgeon to Guy's Hospital.

The Throat Hospital, Gordon Square, has received a legacy of £1,000 from the late Mrs. Ellen Harris.

The Medical Congress at Berlin commenced last week. Some interesting papers were read, and discussions followed. The discussion on Professor Lubrich's paper on "Tuberculosis," wherein he deprecated Dr. Koch's methods, was very warmly debated.

On the 20th inst. the annual parade of the corps of Commissionaires will take place at the Chelsea Barracks, when a collection in aid of the Prince of Wales's Hospital Fund will be made. Miss Alice Glyn gave a mandoline concert in aid of the same Fund this week, in the Steinway Hall. Miss Glyn was assisted by Mdlle. Soldi, Signore Caprile, and Zardo and her amateur circle of mandoline and guitar players.

As a memorial of the Diamond Jubilee of Her Majesty the Queen, the Committee of Management of the Hospital for Consumption and Diseases of the Chest, Brompton, are making a special appeal to the subscribers and friends of the charity on behalf of the proposed country branch and convalescent home, the medical staff being unanimous in considering that such an addition should be established.

We much regret to announce the death of Captain Storrar-Smith, secretary of the City of London Hospital for Diseases of the Chest. He had not been well for some time previous to his death, which took place after a few days' illness on Whit Sunday. Captain Storrar-Smith was previously an officer in the commissariat department of the army. He entered the services of the Hospital in 1880, therefore filling his post for 17 years, and was an ideal secretary, amiable, courteous and unselfish; and his loss is severely felt by the committee and hospital staff, by whom he was held in the highest esteem. Captain Storrar-Smith was 56 years of age and unmarried.

Although the plague in Bombay is well-nigh over, fresh outbreaks in various districts still occur. The introduction and spread of the disease is clearly traceable to the evasion of regulations and precautions.

The new Cholera Hospital for Cardiff at Flat Holm Island is now completed. The buildings consist of a pavilion, laundry block, and a crematorium. There are two wards of twelve beds. The cost has been £3,000.

Sir J. Blundell Maple, amongst other provisions for the welfare of his employés, has now provided for them a Convalescent Home at Harpenden. Accommodation is provided for twenty patients, eight male, twelve female. Reading, writing, conversation, and private sitting rooms are included in arrangements which secure every comfort to the occupants of the Home.

We learn that the hospital at Llanelly has been entirely closed, and all the patients have been removed in cabs to their homes or other dwelling-houses, in consequence of an outbreak of scarlet fever having occurred in the hospital. Three mild cases are being treated, and the hospital has been closed for a fortnight, but it is hoped that the accident ward will be ready in a few days. The institution is being thoroughly disinfected, and every precaution is taken to prevent a spread of the fever, the occurrence of which had caused considerable excitement in the town.

Italy up to the present time has permitted foreign medical men to practise within the kingdom without an Italian diploma, but upon foreign patients only. A movement is now on foot to further restrict the practice, and the subject is under consideration of Parliament. Already a meeting of English doctors has been held, and a deputation has visited the Under Secretary of State for the Interior to point out how the proposed new law would inflict serious injury on English doctors long established in Italy, and possibly influence the influx of English visitors. Whilst the matter is under consideration the General Medical Council of England will probably, at Dr. Eyre's request, address the Italian Under Secretary of State, urging that the reciprocity clause of the Medical Act of 1886 be adopted between England and Italy.

Owing to the publication of an annotation in our columns last week, entitled "A Jubilee Eviction," and referring to the Royal Eye Hospital, Southwark, the following appeared in the *Star* newspaper: "A *Star* man went down to the hospital in St. George's Circus this morning to learn if this could be true. He found the secretary in his office surrounded by timber and plans for seating, while more timber was being carried in by the British workman. Neither nurses nor patients were visible. The secretary read The Hospital article through, and then replied—'All I can say is we are glad to have so good an advertisement. We do not answer The Hospital.' 'But are the facts as stated?' inquired the *Star* man. 'I have nothing to say on the matter at all,' was the reply, 'save that it is a good advertisement for us.' And then somebody else turned up—to hire a Jubilee seat—and the secretary turned to his requirements."

